# Postmortem concentrations of ropivacaine, bupivacaine, and lidocaine in femoral venous blood after hip fracture surgery

**DOI:** 10.1007/s00414-023-03000-6

**Published:** 2023-04-19

**Authors:** Petteri Oura, Antti Virtanen, Juho Nurkkala, Pirkko Kriikku, Ilkka Ojanperä

**Affiliations:** 1 Forensic Medicine Unit, Finnish Institute for Health and Welfare, P.O. Box 30, 00271 Helsinki, Finland; 2 Department of Forensic Medicine, Faculty of Medicine, University of Helsinki, P.O. Box 21, 00014 Helsinki, Finland; 3 Department of Emergency Medicine and Services, Helsinki University Hospital, P.O. Box 340, 00029 Helsinki, Finland; 4 Forensic Toxicology Unit, Finnish Institute for Health and Welfare, P.O. Box 3, 00271 Helsinki, Finland

**Keywords:** Hip fracture, Surgery, Postoperative, Local anesthetic, Postmortem, Toxicology, Forensic, Medico-legal

## Abstract

Pain relief in hip fracture patients may be sought by injecting local anesthetic such as ropivacaine, bupivacaine, and lidocaine to the femoral area. As femoral veins are a routine sampling site for postmortem blood, this short report aimed to describe the levels of local anesthetics in ipsilateral (i.e., side of surgery) and contralateral (i.e., opposite side) femoral blood in ten medico-legal autopsy cases that had undergone a hip fracture surgery within 7 days before death. Postmortem blood samples were systematically collected from the ipsilateral and contralateral femoral veins, and toxicological analysis was performed in an accredited laboratory. The sample comprised six female and four male decedents who died at the age of 71–96 years. Median postoperative survival was 0 days and median postmortem interval 11 days. Strikingly, ropivacaine concentration was a median of 24.0 (range 1.4–28.4) times higher on the ipsilateral than contralateral side. The median ipsilateral concentration of ropivacaine clearly exceeded the 97.5th reference percentile measured in this laboratory for ropivacaine in postmortem cases representing all causes of death. The remaining drugs did not show high concentrations or notable differences between the sides. Our data clearly advise against performing postmortem toxicology on femoral blood from the operated side; the contralateral side may constitute a better sampling site. Toxicology reports that are based on blood collected from the operated area should be interpreted with caution. Larger studies are needed to confirm the findings, with accurate records of the dosage and administration route of local anesthetics.

**Supplementary Information:**

The online version contains supplementary material available at 10.1007/s00414-023-03000-6.

## Introduction

Hip fracture (i.e., proximal femoral fracture) is a debilitating injury that primarily occurs in ageing individuals [1]. The incidence is highest in the Western world (Europe, Russia, Northern America, and Australia), with more than 150 fractures per 100,000 individuals annually [2]. As surgical treatment is generally required [3], hip fracture repair is among the most common emergency procedures in orthopedics [4]. Hip fractures are associated with high mortality [1, 3] and thus carry medico-legal significance.

As hip fractures and surgical treatment are associated with a considerable amount of pain, patients require sufficient pain relief [5, 6]. Regional anesthesia includes peripheral and neuraxial blocks, i.e., injection of local anesthetic around peripheral nerves or close to the spinal cord, respectively [4]. Fascia iliaca compartment block and femoral nerve block are the two most commonly performed peripheral blocks to hip fracture patients [7]; both methods involve injection of local anesthetic in close proximity to the femoral vein. Nerve blocks may be induced by a single injection, repetitive doses, or continuous infusion. General anesthesia, if needed, is also often supplemented with nerve blocks.

Ropivacaine, bupivacaine, and lidocaine are amide local anesthetics synthesized in the 1900s [8]. They reduce sodium ion permeability in nerve cell membranes, reversibly blocking nerve impulse conduction [9]. Main uses include infiltration and regional anesthesia; ropivacaine and bupivacaine are long-acting while lidocaine is a short-acting agent.

Medico-legal autopsy may be requested to decipher whether a hip fracture patient’s death is primarily attributed to the injury, a surgical or medical adverse event, or an underlying natural cause. The autopsy is often supplemented with toxicology, femoral veins being a routine sampling site for postmortem blood [10–12]. However, femoral blood levels of local anesthetics have not previously been characterized among decedents who have undergone a recent hip fracture surgery. This short report aimed to fill the gap in the literature, accounting for both ipsilateral and contralateral femoral blood. These data could be exploited to inform, for example, choice of optimal sampling site and interpretation of toxicology results among medico-legal cases with a recent hip fracture surgery.

## Materials and methods

### Protocol

In Finland, medico-legal autopsies are performed at the regional offices of the Forensic Medicine Unit, Finnish Institute for Health and Welfare, with 8000 to 9000 autopsies per year [13]. Data collection for this study was performed at the Forensic Medicine Unit and Forensic Toxicology Unit, Finnish Institute for Health and Welfare, from April 2020 to July 2022. The sample comprised ten consecutive autopsy cases that had undergone a recent hip fracture surgery and were autopsied by a board-certified forensic pathologist (A.V.) at the Helsinki office of the Forensic Medicine Unit. Additional inclusion criteria were as follows: the diagnosis was femoral neck, intertrochanteric, or subtrochanteric fracture at either hip; the surgical procedure was total arthroplasty, hemiarthroplasty, or internal fixation; and the operation occurred ≤ 7 days before death.

Toxicological data were retrieved from the forensic toxicology database maintained by the Finnish Institute for Health and Welfare that contains results from all postmortem toxicological analyses performed in connection with a medico-legal investigation. The data retrieval grounded on the research permit THL/1922/6.02.00/2017 issued by the Finnish Institute for Health and Welfare, Finland. According to the Finnish legislation, no ethical approval is needed for studies on de-identified register-based data (Personal Data Act 523/1999).

### Background characteristics

Data on sex (male/female), age at death (years), side of hip surgery (left/right), and postoperative survival (i.e., time from the operation to death in full days) were obtained from police reports and autopsy referrals. Postmortem interval (i.e., time from death to autopsy in full days) was calculated on the basis of the time of death and date of autopsy. At the autopsy, height and weight were measured using a mechanical height rod and digital scale, and postmortem body mass index (kg/m^2^) was calculated as weight in kilograms divided by height in meters squared. Manner of death was determined by the forensic pathologist in accordance with the circumstances and primary cause of death.

### Postmortem toxicology

The autopsy involved systematic sampling of postmortem femoral venous blood from both the ipsilateral and contralateral sides. Ipsilateral refers to the side of surgery and contralateral to the opposite. Blood samples were stored in tubes containing sodium fluoride as a stabilizing agent and stored refrigerated until analysis.

The postmortem toxicological examination included the screening and quantification of hundreds of drugs by quality-controlled methods as described in detail elsewhere [12]. Specifically, ropivacaine, bupivacaine, and lidocaine were screened and quantified in postmortem femoral blood using ultra-high performance liquid chromatography coupled to photodiode array and charged aerosol detection [14]. The limit of quantification was 0.05 mg/L for all three analytes.

The 95th and 97.5th reference percentiles for ropivacaine, bupivacaine, and lidocaine concentrations were obtained from a comprehensive Finnish report [12]. The library of reference concentrations was based on an 18-year dataset in a national library at the Forensic Toxicology Unit, Finnish Institute for Health and Welfare, representing all causes of death (*n* = 122,234 cases).

### Statistical analysis

Data analysis was performed in SPSS version 27 (IBM, Armonk, NY). Bar charts and scatter plots were drawn in Excel version 2005 (Microsoft, Redmond, WA). Descriptive statistics were presented as frequencies and percentages for categorical variables, and as medians and ranges (minimum − maximum value) for continuous variables. Medians and ranges were used due to the small sample size. In addition to presenting median, maximum, and minimum concentrations of ropivacaine, bupivacaine, lidocaine, and other drugs on the ipsilateral and contralateral sides, an ipsilateral-to-contralateral ratio was calculated. Bar charts were used to illustrate median, maximum, and minimum concentrations among the sample. Finally, the association of ropivacaine concentration with background variables was illustrated by scatter plots; numeric comparisons were not performed due to the small sample size.

## Results

Background characteristics of the sample are presented in Table 1. The sample comprised ten decedents, of whom six were women and four men, aged 71–96 years. Median postoperative survival was 0 days and median postmortem interval 11 days. The manner of death was declared accident in six cases and natural in four cases; none of the deaths were due to medication error, intoxication, or surgical or medical adverse event.

**Table 1:** Background characteristics of the sample (*n* = 10)

Characteristic	Percentage (*n*)	Median (range)
Sex		
Male	40 (4)	
Female	60 (6)	
Age, years		88 (71–96)
Body mass index, kg/m^2^		22.1 (14.4–25.8)
Side of hip surgery		
Left	70 (7)	
Right	30 (3)	
Postoperative survival, days		0 (0–5)
Postmortem interval, days		11 (4–15)
Manner of death		
Accident	60 (6)	
Natural	40 (4)	

**Fig. 1:**
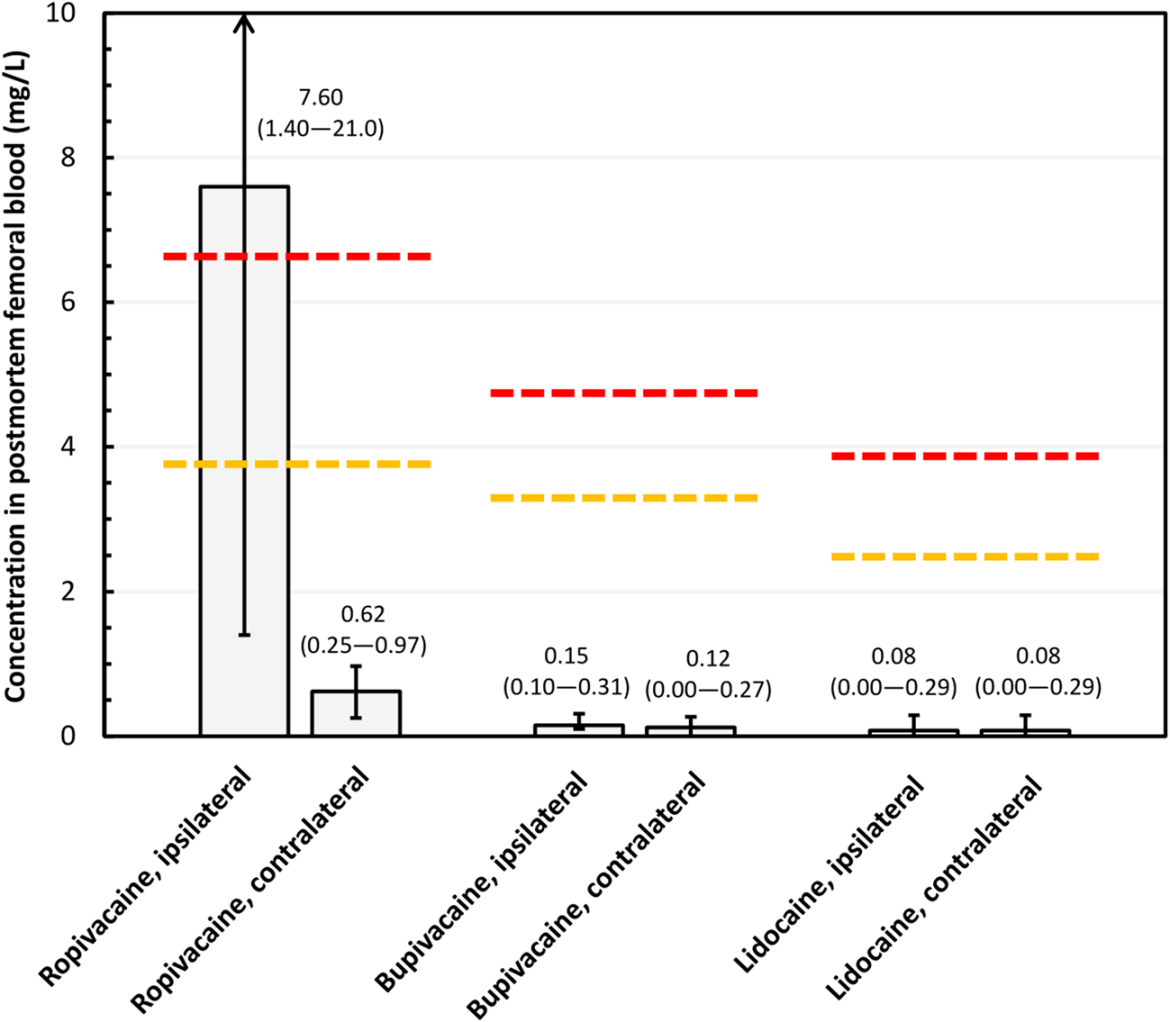
Postmortem concentrations of ropivacaine, bupivacaine, and lidocaine in ipsilateral and contralateral femoral blood among cases that were positive for the respective agent (ropivacaine, *n* = 5; bupivacaine, *n* = 4; lidocaine, *n* = 5). Ipsilateral refers to the side of surgery and contralateral to the opposite. Boxes are medians and whiskers indicate minimum and maximum values. Yellow and red dashed lines correspond to the 95th and 97.5th reference percentiles measured in this laboratory for ropivacaine, bupivacaine, and lidocaine in postmortem cases representing all causes of death [12]

Figure 1 illustrates the concentrations of ropivacaine, bupivacaine, and lidocaine in postmortem femoral blood; ipsilateral and contralateral concentrations are illustrated separately. Table 2 presents the numerical concentration data, as well as the ratios between ipsilateral and contralateral concentrations. Supplementary Table 1 presents the other drugs that were detected in routine toxicology. Of the sample, five decedents were positive for ropivacaine, four for bupivacaine, and five for lidocaine. An additional 22 other drugs were detected among the sample.

**Table 2:** Concentrations of ropivacaine, bupivacaine, and lidocaine in postmortem femoral blood among cases that were positive for the respective agent

Agent	Ipsilateral concentration (mg/L)	Contralateral concentration (mg/L)	Ratio of concentrations
Ropivacaine (*n* = 5)			
#1	1.4	0.97	1.44:1
#2	7.6	0.62	12.3:1
#3	6.0	0.25	24.0:1
#4	10	0.40	25.0:1
#5	21	0.74	28.4:1
Bupivacaine (*n* = 4)			
#1	0.10	–	**–**
#2	0.17	0.15	1.13:1
#3	0.31	0.27	1.15:1
#4	0.12	0.089	1.35:1
Lidocaine (*n* = 5)			
#1	–	0.077	**–**
#2	0.067	–	**–**
#3	0.079	–	**–**
#4	0.29	0.29	1.00:1
#5	0.15	0.14	1.07:1

Ipsilateral refers to the side of surgery and contralateral to the opposite

The concentrations of bupivacaine and lidocaine were well below the 95th and 97.5th reference percentiles measured in this laboratory for bupivacaine and lidocaine in postmortem cases representing all causes of death, with no notable differences between the ipsilateral and contralateral sides (Fig. 1, Table 2). In contrast, ropivacaine concentration was a median of 24.0 times (range 1.4–28.4 times) higher on the ipsilateral side than the contralateral side (Table 2). The median ipsilateral concentration of ropivacaine clearly exceeded both the 95th and 97.5th reference percentiles measured in this laboratory for ropivacaine representing all causes of death (Fig. 1). The remaining drugs did not show notable differences between the sides (Supplementary Table 1).

Supplementary Figs. 1–3 show the ipsilateral ropivacaine concentration, contralateral ropivacaine concentration, and the ratio between ipsilateral and contralateral concentrations in relation to the background variables. The length of postoperative survival appeared to influence ropivacaine positivity, as all the cases that were positive for ropivacaine in postmortem toxicology had a postoperative survival of 0 days. The remaining variables (i.e., sex, age, body mass index, postmortem interval, side of surgery, and manner of death) did not appear to influence ropivacaine concentration in a systematic manner.

## Discussion

This short report is among the first to describe postmortem levels of local anesthetics in femoral venous blood after hip fracture surgery. Strikingly, ropivacaine concentration was a median of 24 times higher on the operated side than on the contralateral side, clearly exceeding the 97.5th reference percentile measured in this laboratory for ropivacaine in postmortem cases representing all causes of death. Lidocaine, bupivacaine, and other drugs did not show high concentrations or notable differences between the sides. The distribution of ropivacaine in the body was therefore clearly different from the other local anesthetics; this may be explained by their different usage.

Even though based on a small dataset, the finding of high ipsilateral ropivacaine concentration is expected to have value in the medico-legal context. In spite of the seemingly high concentrations, none of the deaths were caused or contributed to by a medication error, intoxication, or surgical or medical adverse event. As such, toxicology reports that are based on blood from close proximity to the operated area should be interpreted with caution. Our data clearly advise against performing postmortem toxicology on femoral blood from the operated side; the contralateral side may constitute a better sampling site.

Although our data are limited in terms of extracting specific mechanisms for the findings, we propose that ropivacaine may not have diffused from the injection site to the rest of the body, unlike the other local anesthetics. This would explain the differences between local anesthetics, as well as the difference between ipsilateral and contralateral levels of ropivacaine. The two most commonly used peripheral nerve blocks (fascia iliaca compartment and femoral nerve block) involve injecting a relatively large volume of local anesthetic in the soft tissue near the femoral vein [7]; in the Helsinki region, these blocks mainly use ropivacaine. Local guidelines recommend boosting ropivacaine with dexamethasone adjuvant which prolongs block duration by several hours. The underlying mechanism is not fully understood, but there is some evidence suggesting that dexamethasone might cause local vasoconstriction in the tissue level, slowing the absorption of the sedative agent [15]. In our region, lidocaine and bupivacaine are rarely used in a similar manner to ropivacaine, which would explain why lidocaine and bupivacaine did not show notable differences between the sides. The physico-chemical and pharmacokinetic parameters of the three local anesthetics are too similar to explain the difference in the behavior of ropivacaine compared to the others. According to the INXBASE interaction database [16], none of the three local anesthetics are known to have clinically significant interactions with the concomitant drugs found in the respective cases.

As for the background variables, postoperative survival appeared to influence ropivacaine positivity, as all the deceased that were positive for ropivacaine died within a day after the surgery. In our data, the remaining variables (i.e., sex, age, body mass index, postmortem interval, side of surgery, and manner of death) did not appear to have systematic associations with ropivacaine concentration. However, we acknowledge that our dataset was small for making precise inferences regarding the association between background variables and drug concentrations.

Hip fracture surgery is already among the most common emergency orthopedic procedures [4], and the incidence of hip fractures is expected to further increase steeply [1], leading to increased operation numbers. Current guidelines strongly advocate preoperative femoral or fascia iliaca block instead of opioids for all hip fracture patients [17, 18]. Mortality remains relatively high in both short and long term [1, 3]. A forensic pathologist may be requested to evaluate whether a patient’s death was primarily due to the hip fracture or an underlying natural cause, and there may also be questions regarding the possibility of a surgical or medical adverse event. The present data will aid forensic pathologists choose optimal blood sampling site and interpret toxicology results accordingly.

The report has several strengths. Consecutive cases fulfilling the inclusion criteria were selected from the routine case flow. The sampling of femoral venous blood systematically involved both left and right sides. Comprehensive toxicological analysis was performed in an accredited national laboratory; other drugs detected in routine toxicology did not show notable differences between the sides. A number of background variables were collected and correlated against toxicological findings, including sex, age, body mass index, postoperative survival, and postmortem interval. However, some limitations should also be noted. As the present report was based on a sample of ten individuals aged ≥ 71 years, larger studies are needed to confirm the findings and investigate other age groups. Future studies should also incorporate data on the dosage and administration route of local anesthetics and possible adjuvants, as these were not available to us.

## Conclusion

In medico-legal autopsy cases with a recent hip fracture surgery, ropivacaine concentration in femoral venous blood was manifold on the operated side compared to the contralateral side. We speculate that ropivacaine may not have diffused from the injection site to the rest of the body, unlike the other local anesthetics; this may be explained by their different usage. Our findings strongly suggest that postmortem blood sampling should not be performed on the ipsilateral side. Moreover, particular caution should be exercised when interpreting toxicology reports that involve sample material obtained near the operated area. In the future, larger studies are needed to confirm the findings and investigate other age groups, with accurate records of the dosage and administration route of local anesthetics and possible adjuvants.

## Supplementary Information

Below is the link to the electronic supplementary material.

Supplementary file 1 (DOCX 1387 KB)

Supplementary file 2 (DOCX 56 KB)

## Data Availability

The dataset used in this report is not made publicly available due to local privacy regulations.
