# Supplementary material for: Postmortem concentrations of ropivacaine, bupivacaine, and lidocaine in femoral venous blood after hip fracture surgery
Source: Int J Legal Med. 2023 Apr 19;137(4):1071–6. doi: 10.1007/s00414-023-03000-6 (PMC10247554; doi:10.1007/s00414-023-03000-6)
Supplement: Supplementary file 1 — (DOCX 1387 KB) [file 414_2023_3000_MOESM1_ESM.docx]

**SUPPLEMENTARY FIGURES**


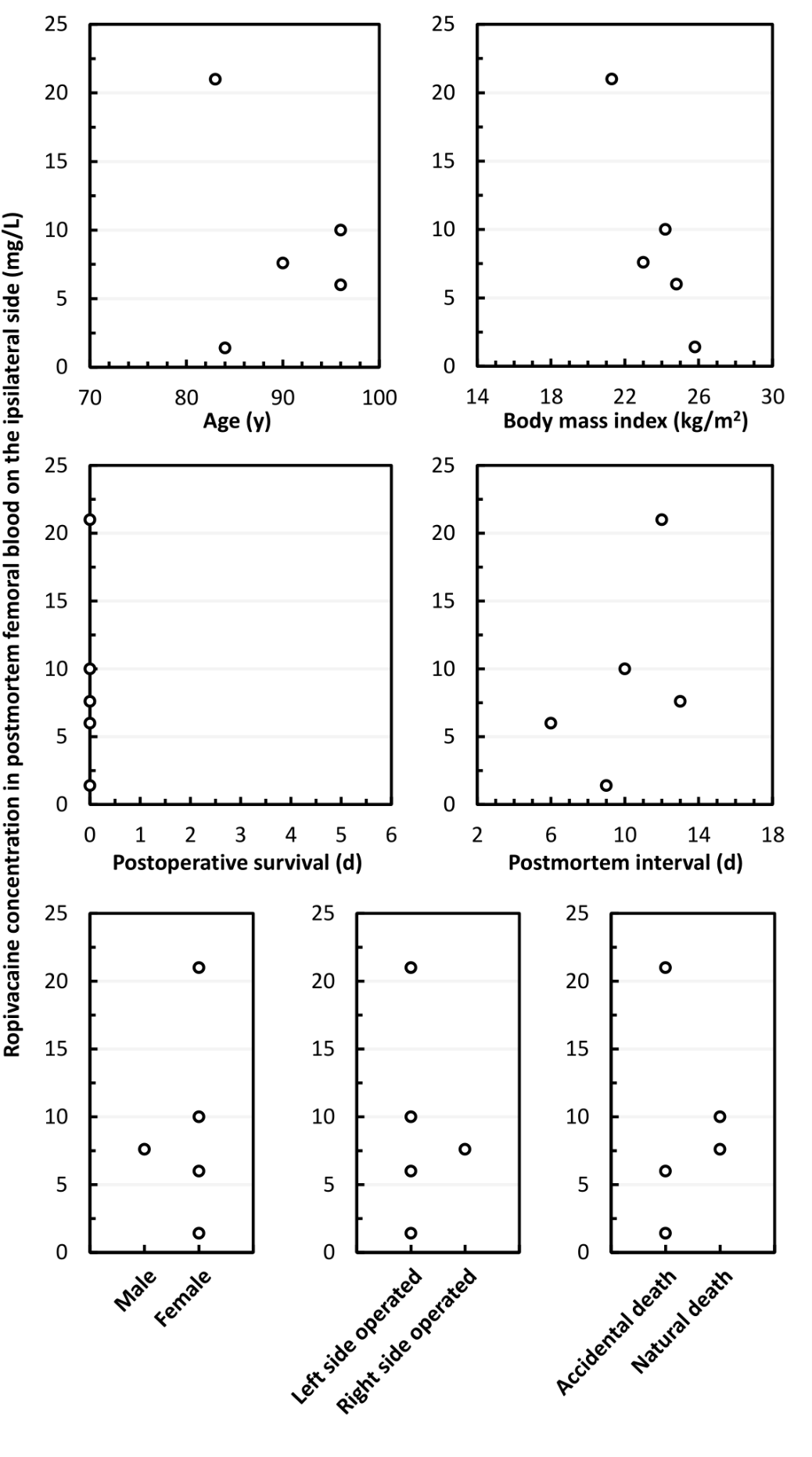
 **Supplementary Figure 1**. Scatter plots illustrating the relationship between ipsilateral ropivacaine concentration (i.e., side of surgery) and background variables among cases that were positive for ropivacaine (n = 5).


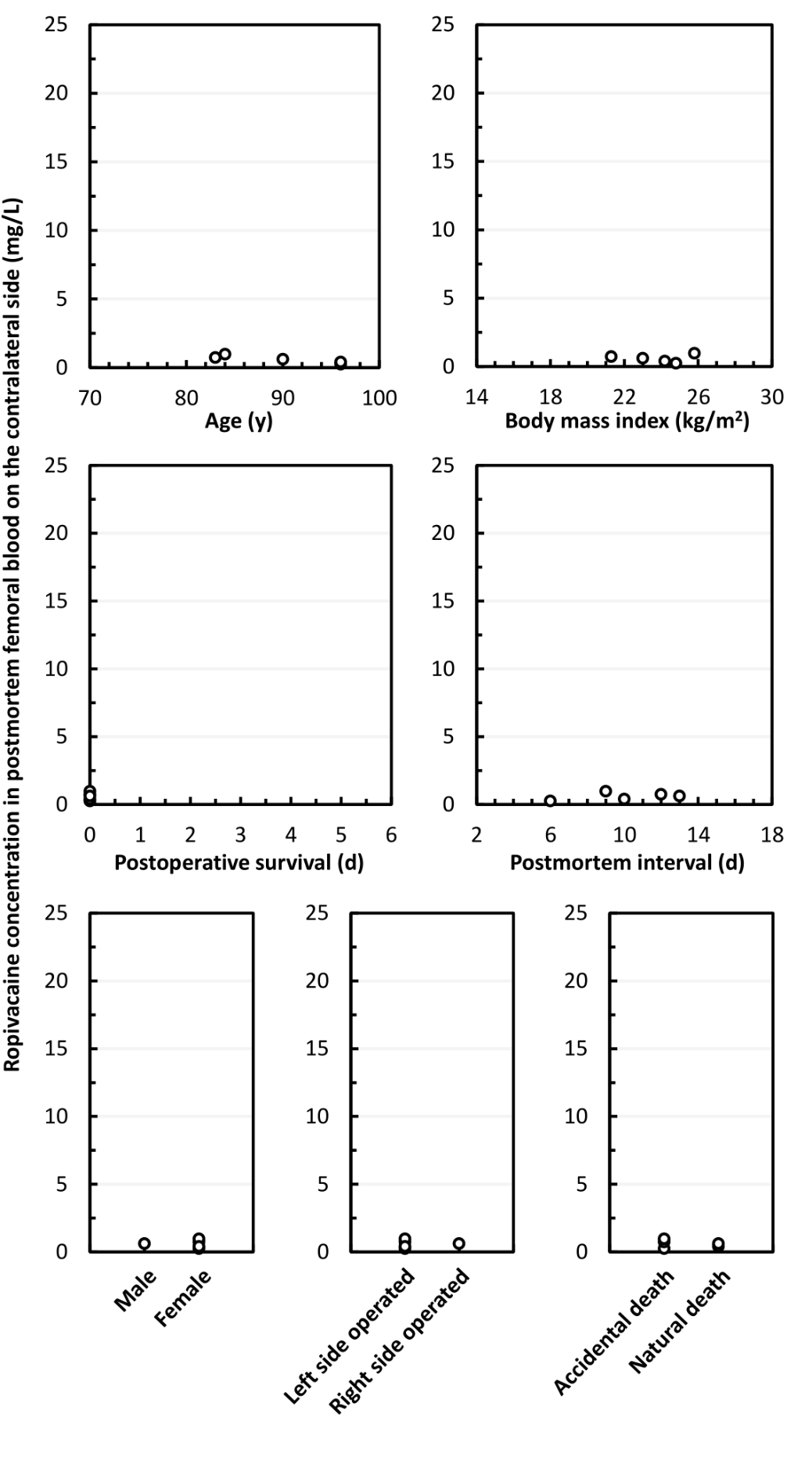


**Supplementary Figure 2**. Scatter plots illustrating the relationship between contralateral ropivacaine concentration (i.e., opposite side to surgery) and background variables among cases that were positive for ropivacaine (n = 5).


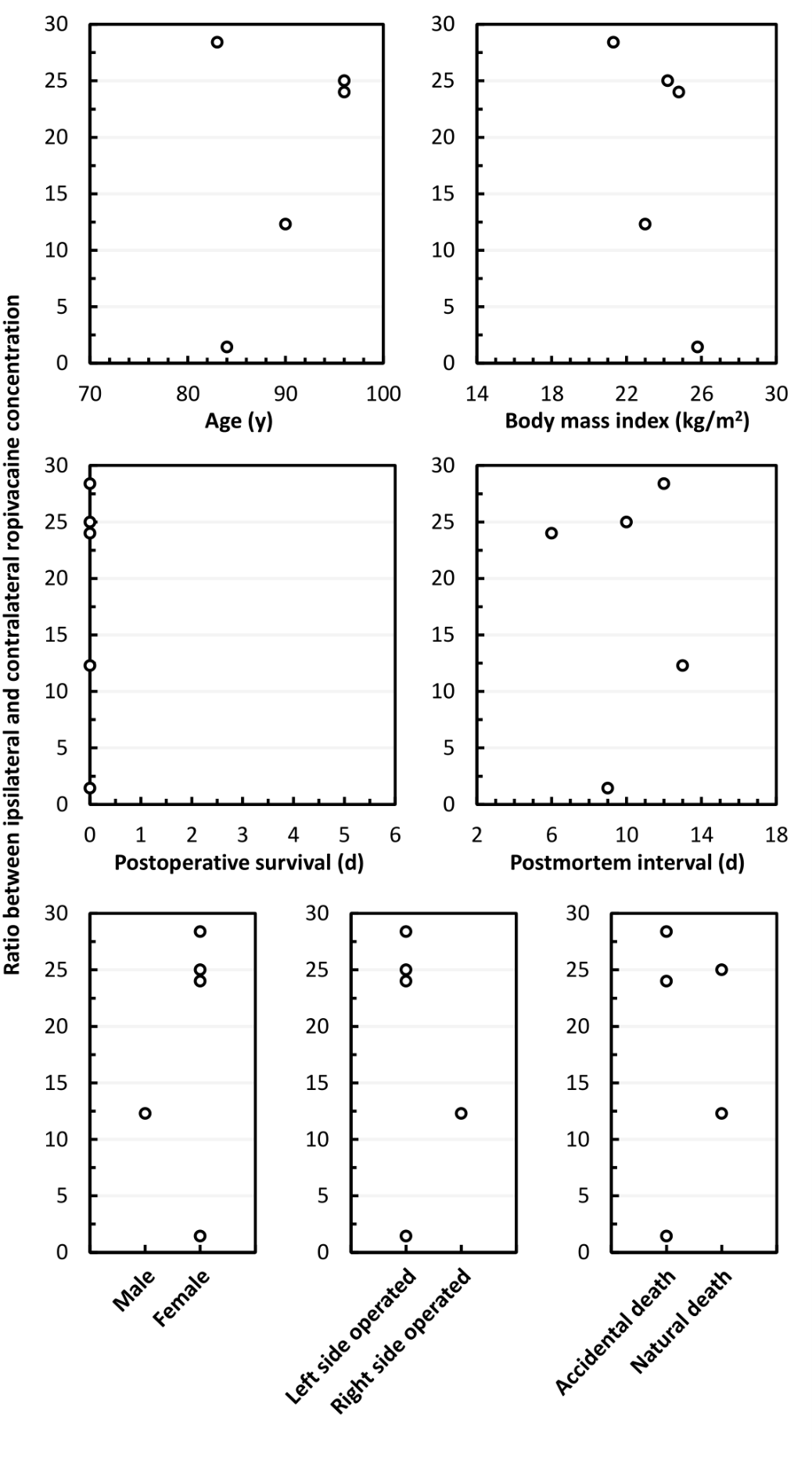


**Supplementary Figure 3**. Scatter plots illustrating the relationship between ropivacaine concentration ratio (ipsilateral to contralateral) and background variables among cases that were positive for ropivacaine (n = 5). Ipsilateral refers to the side of surgery and contralateral to the opposite.
