# Supplementary material for: Postmortem concentrations of ropivacaine, bupivacaine, and lidocaine in femoral venous blood after hip fracture surgery
Source: Int J Legal Med. 2023 Apr 19;137(4):1071–6. doi: 10.1007/s00414-023-03000-6 (PMC10247554; doi:10.1007/s00414-023-03000-6)
Supplement: Supplementary file 2 — (DOCX 56 KB) [file 414_2023_3000_MOESM2_ESM.docx]

**SUPPLEMENTARY TABLES**

**Supplementary Table 1**. Concentrations of other drugs in postmortem femoral blood among cases that were positive for the respective agent.

| Agent | Ipsilateral  concentration (mg/L) | Contralateral concentration (mg/L) | Ratio of concentrations |
| --- | --- | --- | --- |
| Acetaminophen (n = 9) |  |  |  |
| #1 | 12 | - | **-** |
| #2 | 5.4 | 10 | **0.54 : 1** |
| #3 | 5.9 | 8.8 | **0.67 : 1** |
| #4 | 6.6 | 10 | **0.66 : 1** |
| #5 | 22 | 25 | **0.88 : 1** |
| #6 | 12 | 13 | **0.92 : 1** |
| #7 | 18 | 18 | **1.00 : 1** |
| #8 | 47 | 47 | **1.00 : 1** |
| #9 | 24 | 23 | **1.04 : 1** |
| Alfentanil (n = 1) |  |  |  |
| #1 | 8.3 E-3 | 9.1 E-3 | **0.91 : 1** |
| Bisoprolol (n = 1) |  |  |  |
| #1 | <0.1 | <0.1 | **-** |
| Buprenorphine (n = 1) |  |  |  |
| #1 | - | 0.53 E-3 | **-** |
| Buprenorphine, nor- (n = 1) |  |  |  |
| #1 | - | 0.59 E-3 | **-** |
| Caffeine (n = 1) |  |  |  |
| #1 | 3.2 | 3.2 | **1.00 : 1** |
| Furosemide (n = 3) |  |  |  |
| #1 | - | <1.0 | **-** |
| #2 | <1.0 | <1.0 | **-** |
| #3 | 2.5 | - | **-** |
| Galantamine (n = 1) |  |  |  |
| #1 | 0.12 | 0.13 | **0.92 : 1** |
| Haloperidol (n = 1) |  |  |  |
| #1 | 0.003 | 0.004 | **0.75 : 1** |
| Haloperidol, hydroxy- (n = 1) |  |  |  |
| #1 | 0.006 | 0.007 | **0.86 : 1** |
| Hydroxychloroquine (n = 1) |  |  |  |
| #1 | 1.4 | 1.4 | **1.00 : 1** |
| Lorazepam (n = 1) |  |  |  |
| #1 | <0.005 | <0.005 | **-** |
| Memantine (n = 2) |  |  |  |
| #1 | 0.47 | 0.48 | **0.98 : 1** |
| #2 | 0.72 | 0.65 | **1.11 : 1** |
| Mirtazapine (n = 2) |  |  |  |
| #1 | <0.1 | <0.1 | **-** |
| #2 | <0.1 | <0.1 | **-** |
| Ondansetron (n = 1) |  |  |  |
| #1 | 0.024 | 0.026 | **0.92 : 1** |
| Oxazepam (n = 1) |  |  |  |
| #1 | 0.16 | 0.14 | **1.14 : 1** |
| Oxycodone (n = 4) |  |  |  |
| #1 | 0.04 | 0.05 | **0.80 : 1** |
| #2 | 0.013 | 0.013 | **1.00 : 1** |
| #3 | 0.054 | 0.052 | **1.04 : 1** |
| #4 | 0.017 | 0.016 | **1.06 : 1** |
| Oxycodone, nor- (n = 4) |  |  |  |
| #1 | 0.004 | 0.005 | **0.80 : 1** |
| #2 | 0.029 | 0.03 | **0.97 : 1** |
| #3 | 0.03 | 0.03 | **1.00 : 1** |
| #4 | 0.012 | 0.011 | **1.09 : 1** |
| Oxymorphone (n = 1) |  |  |  |
| #1 | - | <0.003 | **-** |
| Propofol (n = 1) |  |  |  |
| #1 | 0.04 | 0.03 | **1.33 : 1** |
| Rivaroxaban (n = 2) |  |  |  |
| #1 | 0.11 | 0.095 | **1.16 : 1** |
| #2 | 0.25 | - | **-** |
| Temazepam (n = 2) |  |  |  |
| #1 | <0.02 | - | **-** |
| #2 | 0.13 | 0.13 | **1.00 : 1** |

Ipsilateral refers to the side of surgery and contralateral to the opposite.
